# The Natural history of Epicardial Adipose Tissue Volume and Attenuation: A long-term prospective cohort follow-up study

**DOI:** 10.1038/s41598-020-63135-z

**Published:** 2020-04-28

**Authors:** Nitesh Nerlekar, Udit Thakur, Andrew Lin, Ji Quan Samuel Koh, Elizabeth Potter, David Liu, Rahul G. Muthalaly, Hashrul N. Rashid, James D. Cameron, Damini Dey, Dennis T. L. Wong

**Affiliations:** 1 Monash Cardiovascular Research Centre, Monash University and MonashHeart, Monash Health, Clayton, Victoria, Australia; 2 Biomedical Imaging Research Institute, Cedars Sinai Medical Center, Los Angeles, CA USA

**Keywords:** Cardiology, Angiogenesis

## Abstract

Epicardial adipose tissue (EAT) is associated with cardiovascular risk. The longitudinal change in EAT volume (EATv) and density (EATd), and potential modulators of these parameters, has not been described. We prospectively recruited 90 patients with non-obstructive coronary atherosclerosis on baseline computed tomography coronary angiography (CTCA) performed for suspected coronary artery disease to undergo a repeat research CTCA. EATv in millilitres (mL) and EATd in Hounsfield units (HU) were analysed and multivariable regression analysis controlling for traditional cardiovascular risk factors (CVRF) performed to assess for any predictors of change. Secondary analysis was performed based on statin therapy. The median duration between CTCA was 4.3years. Mean EATv increased at follow-up (72 ± 33 mL to 89 ± 43 mL, p < 0.001) and mean EATd decreased (baseline −76 ± 6 HU vs. −86 ± 5 HU, p < 0.001). There were no associations between baseline variables of body mass index, age, sex, hypertension, hyperlipidaemia, diabetes or smoking on change in EATv or EATd. No difference in baseline, follow-up or delta EATv or EATd was seen in patients with (60%) or without baseline statin therapy. In this select group of patients, EATv consistently increased and EATd consistently decreased at long-term follow-up and these changes were independent of CVRF, age and statin use. Together with the knowledge of strong associations between EAT and cardiac disease, these findings may suggest that EAT is an independent parameter rather than a surrogate for cardiovascular risk.

## Introduction

Epicardial adipose tissue (EAT) has been described to associate with coronary artery disease^1^ as well as influence myocardial function and geometry^2^. It has been suggested that vasocrine or paracrine effects may be the intermediary for transmission of pro-inflammatory adipokines from dysfunctional adipose tissue to the adjacent myocardium or coronary vasculature^3^. Additionally, local compressive forces of excess EAT may result in reduced myocardial compliance and subsequent diastolic dysfunction. However, most studies are cross-sectional in nature and the natural history of EAT is not well described. The few published studies are limited to small cohorts of either asymptomatic patients undergoing cardiac screening^4,5^, or elevated-risk patients either after an acute coronary syndrome, or with the presence of high risk coronary plaque characteristics^6^. EAT is best evaluated by volumetric measurement on computed tomography (EAT)^7^, a non-invasive radiography modality designed for use in low-intermediate risk symptomatic patients. As EAT is universal to human anatomy, it is important to evaluate its natural evolution in this cohort to better understand what cardiovascular risk factors may influence its change, as it thus far remains simply an associative marker of cardiac risk that is thought to be modulated by other metabolic markers or obesity measures. As EAT has been increasingly described as a marker of adipose tissue activity and inflammation which can be gauged by the attenuation of fat, the density of EAT is also of interest to investigate as an alternative marker of risk beyond the total volume of EAT alone^8^. The long-term natural history of EAT has not been assessed in general cohorts of suspected coronary artery disease that comprise the vast majority of patients undergoing coronary assessment on CT coronary angiography, most of whom will have non-obstructive CAD^9^.

Therefore, we sought to prospectively examine the long-term changes in EAT volume and density in a cohort of patients with non-obstructive coronary artery disease only. We also sought to evaluate the potential effect of statin therapy on these markers given the pleiotropic effects of this agent which includes anti-inflammatory potential.

## Methods

Patients were retrospectively identified from a registry of patients who underwent CT coronary angiography at Monash Heart, Monash Health, Melbourne, Australia between 2010–2012. The primary inclusion criteria for all patients was the presence of coronary atherosclerosis in at least 1 coronary segment and no visual diameter stenosis of ≥50% of the lumen. Patients were excluded if they had any previous coronary intervention. Patients were randomly selected from the registry in a consecutive fashion using a random number generator to avoid selection bias. Once the primary inclusion and exclusion criteria was met, they were contacted and invited to return for a research specific coronary CT. In the event of pregnancy or a reduction in glomerular filtration rate (GFR) < 30 mL/min or withdrawal of consent, research CTCA would not be performed. Baseline cardiovascular risk factors, and statin use were obtained from the medical record and patient interview and prospectively recorded at follow-up scan. All follow-up scans were performed between 2015–2018. Written informed consent was obtained in all patients and the study was approved by the local ethics committee (Monash Health Human Research Ethics Committee) with all research performed in accordance with relevant guidelines and regulations.

All efforts were made to match scan parameters, particularly for kV between baseline and follow-up CT and all CT were performed using a 320-row multi-detector CT using previous institutional protocol^10^. Briefly, all studies were performed on a 320-detector row system (AquilionOne, Toshiba Medical Systems, Tokyo, Japan). Nitro-glycerine 400 µg sublingually was administered prior to contrast injection. A bolus of 75 mL of 100% Iohexal (Omnipaque 350) was administered at 6 mL/s followed by a 50 mL normal saline chaser. Scanning was manually triggered when peak contrast enhancement in the left ventricle was observed with no enhancement in the right ventricle. Scans were performed via an axial technique with detector collimation of 320 mm × 0.5 mm and no requirement for table movement due to 16 cm cranio-caudal coverage. Prospective electrocardiographic triggering at 70–85% phase window was performed in all patients. Images were reconstructed with a 512 × 512 matrix, 0.5 mm thick sections and 0.25 mm increments with adaptive iterative dose reduction and standard and asymmetric cone beam reconstruction. Rate control therapy was used to aim for acquisition heart rate <65bpm.

Measurement of EAT was performed according to previously described methods^7^. Briefly, the upper EAT boundary was considered to be the bifurcation of the pulmonary trunk and the lower most portion of the cardiac apex were the last slice of the posterior descending artery was seen. Pericardial contours were manually traced at 5–10 slice intervals with observation for interpolation and adjustment performed if required. Adipose tissue was quantified using threshold of −190 Hounsfield units (HU) and −30 HU. The mean density of EAT was recorded. Inter and intra-observer variability demonstrated excellent correlation with both intra-class correlation coefficients of 0.98 respectively.

### Outcomes

There were two co-primary outcomes. Firstly, the difference in follow-up compared to baseline EAT volume and secondly the difference in follow-up compared to baseline EAT density. A secondary analysis was performed to assess differences in the primary outcomes based on statin use

### Statistical analysis

Analysis was performed using STATA 14/MP (StataCorp Ltd, TX). Categorical variables are presented as number and percentage, and continuous variables as mean with standard deviation, or median inter-quartile range. Normality was assessed visually using histogram plots. Categorical variables were compared using McNemar test for paired observations, and chi-squared test for unpaired observations. Continuous data was compared using the paired t-test. Spearman rank correlation coefficients (rho) were reported for baseline comparison of EAT with independent variables. Ordinary least squares simple and multiple linear regression was used with change in EAT volume and change in EAT density (follow up – baseline) as the outcome variable of interest. Beta-coefficients with standard errors are reported for linear regression. We performed a binary logistic regression for analysis with a change of 10% EAT from baseline^6^ as the outcome variable and report results as odds ratios with 95% confidence intervals on univariable and multivariable regression. For body mass index, the delta value (BMI at second scan – BMI at first scan) as well as percentage change in BMI [(BMI at second scan – BMI at first scan)/BMI at first scan × 100] was used. Inter- and intra-observer agreement was evaluated by the intra-class correlation coefficient. A two-tailed p < 0.05 was considered statistically significant.

## Results

There were 100 patients initially identified with 90 patients included in the final analysis. Of the 10 excluded patients, 3 withdrew consent, 3 had GFR < 30 mL/min, and 4 had image quality that was suboptimal for adequate evaluation of EAT or coronary artery disease on the baseline CT scan. None of the identified patients had a significant coronary or cardiac event in the antecedent time period.

Baseline demographics are presented in Table 1. The mean age was 59 ± 11 and 58 (64%) were male patients. There was no difference in traditional risk factors of hypertension, diabetes, dyslipidaemia, smoking or family history of premature coronary disease. There were 54 (60%) of patients on statin therapy which was not different at follow-up. The mean segment involvement score was **3** ± 1.5. The median follow-up duration was 4.3years (IQR 4.1 to 5.5 y, range 3 to 7.8 y).

**Table 1:** Baseline and follow-up demographics in the 90 included patients.

Variable	Baseline	Follow-Up	p-value
Age (years)	59 ± 11	64 ± 9	<0.001
Sex (male)	58 (64%)	—	—
BMI (kg/m^2^)	28.8 ± 6	29.0 ± 6	0.79
Hypertension	50 (56%)	52 (58%)	0.83
Hyperlipidaemia	56 (62%)	64 (71%)	0.37
Family History	56 (62%)	—	—
Smoking	26 (29%)	30 (33%)	0.65
Diabetes	14 (16%)	20 (22%)	0.42

Results are mean ± standard deviation or frequency (%).

BMI – body mass index.

Baseline EATv correlated with baseline BMI (rho = 0.39, p = 0.009) but no other clinical cardiovascular risk factors (Table 2). This relationship remained consistent when all variables were forced into a multivariable regression model (BMI β = 1.935, p = 0.03) (Table 2).

**Table 2:** Univariable correlation and multivariable linear regression between clinical variables and baseline EATv.

Variable	Correlation		Multivariable		
	Spearman rho	p-value	β-coefficient	Standard Error	p-value
Hypertension	0.21	0.16	11.461	9.622	0.241
Smoking	0.07	0.65	2.389	10.779	0.826
Hyperlipidaemia	0.16	0.27	−0.644	10.033	0.949
Family History	0.03	0.86	−3.663	9.858	0.712
Diabetes	0.17	0.26	2.420	13.494	0.859
BMI	0.39	0.009*	1.935	0.857	0.03*
Age	0.29	0.054	0.755	0.436	0.092
Sex	−0.17	0.24	−13.716	9.989	0.178

BMI – body mass index; EATv – epicardial adipose tissue volume; IHD – ischaemic heart disease,

*Denotes p < 0.05.

### Primary outcome

**Figure 1:**
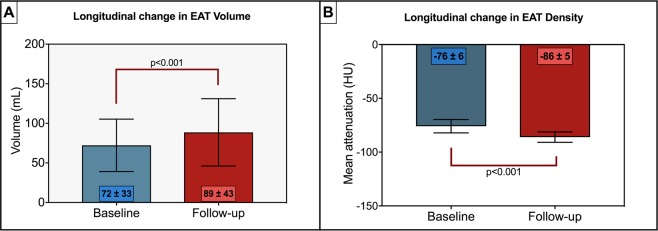
Longitudinal changes in mean EAT volume (**A**) and EAT radiodensity (**B**). Bar graphs with standard deviations demonstrate change in EAT volume and density from baseline to follow-up. EAT – Epicardial Adipose Tissue, HU – Hounsfield Units, mL – millilitres.

Mean EAT volume significantly increased at follow-up (baseline 72 ± 33 mL vs follow-up 89 ± 43 mL, p < 0.001 (Fig. 1A)). Mean EAT density significantly decreased (more negative) over time (baseline −76 ± 6 HU vs. −86 ± 5 HU, p < 0.001) (Fig. 1B).

The mean change in EAT was 16 mL ± 15 mL (range −20mL to 71 mL). Only two patients demonstrated an absolute reduction in EATv. We further analysed EAT change by an increase in 10% of the baseline EAT value which demonstrated that 72 (80%) of patients had an increase of 10% of the baseline EAT.

On assessment restricted to subjects who had an absolute increase in EAT there were no significant associations between clinical variables and increase in EAT at follow-up (Table 3**)**. Increasing age was associated with a reduction in the odds of a greater than 10% change in EAT at a univariable level (OR 0.91 95% CI (0.83–0.99), p = 0.03), however this association was attenuated, and no longer statistically significant in the multivariable logistic regression model (Table 3).

**Table 3:** Multivariable baseline associations of cardiovascular risk factors with delta EATv (absolute difference in EAT at follow-up and baseline), and when EAT modelled as > 10% change compared to baseline.

Variable	Multivariable linear regression (delta EAT volume)			Multivariable logistic regression (EAT >10% baseline)		
	β-coefficient	Standard Error	p-value	OR	95% CI	p-value
Hypertension	−0.128	4.583	0.978	0.67	0.09–5.01	0.69
Smoking	7.176	4.764	0.141	3.77	0.29–49.54	0.31
Hyperlipidaemia	−6.296	4.920	0.209	0.24	0.03–2.36	0.22
Family History	−0.678	4.487	0.881	3.31	0.49–22.38	0.22
Diabetes	8.751	6.106	0.161	3.71	0.21–66.89	0.37
∆ BMI	0.582	0.363	0.118	1.01	0.90–1.14	0.83
Age	0.185	0.193	0.343	0.91	0.81–1.007	0.07
Sex	−6.951	4.396	0.123	1.29	0.17–9.66	0.80

There was no significant association either at univariable or multivariable analysis for EAT density (Table 4). The mean change in EATd was −10 ± 6 HU with 3 patients demonstrating an increase of density over time. Only absolute change in BMI was associated with a change in EATd at a univariable level with increasing change in BMI associated with decreasing (more negative) EATd (beta −0.39, p = 0.008), but this was no longer significant at multivariable analysis (beta −0.16, p = 0.22). No other variables were associated with a change in EATd (Table 4).

**Table 4:** Univariable correlation and multivariable linear regression between clinical variables and baseline EAT density; and delta density.

Variable	Correlation		Multivariable baseline			Multivariable delta EAT density		
	Spearman rho	p-value	β-coefficient	Standard Error	p-value	β-coefficient	Standard Error	p-value
Hypertension	−0.14	0.37	−1.834	2.020	0.370	1.162	1.985	0.562
Smoking	−0.004	0.98	−0.097	2.263	0.966	−0.457	2.149	0.833
Hyperlipidaemia	0.11	0.49	0.798	2.106	0.707	−1.630	2.148	0.453
Family History	0.06	0.68	0.392	2.070	0.851	−0.140	1.936	0.943
Diabetes	0.12	0.45	0.400	2.833	0.889	−2.360	2.746	0.396
BMI^	0.06	0.68	0.148	0.180	0.416	−0.335	0.163	0.074
Age	−0.11	0.49	−0.082	0.092	0.377	−0.028	0.085	0.742
Sex	−0.22	0.16	−3.581	2.097	0.096	1.986	1.940	0.313

^ for change in density, the ∆ BMI was used as the independent variable.

### Effect of statin therapy

There was no difference in baseline EATv by statin use although numerically, statin-taking patients had a higher EATv (statin EATv 76 ± 31 mL vs 66 ± 35 mL, p = 0.29). No significant difference was noted with follow-up EATv by statin group (statin EATv 94 ± 41 mL vs. 81 ± 44 mL, p = 0.15). There was no difference in delta EATv by statin group (statin 17 ± 14 mL vs. 15 ± 17 mL, p = 0.48).

No difference was demonstrated for EATd at baseline or follow-up by statin stratification: baseline statin −76 ± 6 HU vs −77 ± 5 HU, p = 0.34; follow-up −87 ± 5 HU vs. −85 ± 5 HU, p = 0.18) (Table 5) and no predictors of change in density on simple and multivariable regression modelling (data not shown). Inclusion of statin therapy in the multivariable models of EATd or EATv did not result in statistical significance.

**Table 5:** Differences in EAT parameters based on statin use.

EAT parameter	Statin	No Statin	p-value
EATv Baseline	76 ± 31	66 ± 35	0.29
EATv Follow-up	94 ± 41	81 ± 44	0.15
EATd Baseline	−76 ± 6	−77 ± 5	0.34
EATd Follow-up	−87 ± 5	−85 ± 5	0.18

## Discussion

In this select cohort of symptomatic low-risk suspected CAD patients with long-term follow-up and serial CTCA, we demonstrate a consistent increase in absolute EATv and decrease in EATd over time. We also show that there were no significant clinical risk factors that independently associated with longitudinal changes in EATv or EATd. Furthermore, the use of statin therapy did not influence baseline or follow-up values. Coupled with the knowledge that EAT has demonstrated significant associations with cardiac disease, these findings may suggest that EAT is an independent parameter rather than a surrogate for cardiovascular risk.

EAT volume, and more recently attenuation have been increasingly investigated in the literature for associations with cardiac disease^8,11–15^. Several studies have demonstrated significant cross-sectional relationships between EAT and cardiac disease, however a lack of longitudinal data prevents understanding of what therapies or targets may be a modulating factor for EAT.

We noted a significant relationship between EAT and baseline BMI which confirms the findings of other studies that suggest EAT is related to markers of clinical obesity^16^. However, this association was no longer significant after inclusion of other clinical risk factors. This is likely due to confounding from other variables such as hypertension, diabetes and dyslipidaemia that are also strongly related to BMI and mirrors the attenuated relationship of obesity and future coronary disease risk when traditional risk factors are included in multivariable modelling^17^. While several studies have suggested weight loss may result in EAT reduction^18^, these reports are significantly limited by the use of linear thickness measurements of EAT which has been shown to be substandard compared to volumetric measures^7^. There are a few studies that have evaluated serial changes in EATv, independent of effects on other body fat parameters and cardiovascular risk factors. In a study describing the effect of bariatric surgery in severely obese patients, there was a significant reduction in EATv as assessed on magnetic resonance imaging (MRI), however this was not correlated with body fat percentage loss or non-epicardial visceral fat loss^19^. Similar results were seen in a short-term evaluation of exercise testing in obese individuals with serial MRI. Both endurance and resistance training resulted in significant reductions in EATv without significant changes in other cardiometabolic parameters^20^. In another study that evaluated serial non-contrast CT change in EAT in an asymptomatic observational cohort, a greater percentage change in BMI on follow-up was associated with a greater percentage change in EAT^6^. However, this study is significantly different to ours in that only a third of the patients had established atheroma, follow-up CT was incidentally performed rather than mandated by research protocol which may result in selection bias, and there was no change in mean BMI between scans with an absolute difference of only 0.2 kg/m^2^. Our results in addition to all of these findings may support the notion that EAT is distinct from other parameters of body fat distribution and may in fact be differently regulated with a unique physiology.

In a community cohort of 623 asymptomatic Japanese men aged 40–79 years, serial cardiac CT at 4.7 years mean follow up demonstrated a significant increase in EAT volume^5^. The only clinical risk factor associated with change in EATv was current smoking. In our study, smoking did not associate with change in EATv however this may be driven by a very low prevalence (<20%) of smokers in our cohort that combined both ex- and current smokers compared to a 30% prevalence of current and 52% ex-smoker cohort in the aforementioned study. We did note a nominally higher EATv in statin taking patients. The association between statin use and accumulation of organ and body fat has been described^21^, however it remains a subject for further study to evaluate whether statin use may increase EATv but alter EATd, as well as other potential anti-inflammatory agents that have effects on cardiovascular disease^22^. No other studies have described potential predictors of change in EATv on serial prospective CT.

EATd is hypothesised to be a marker of adipose tissue activity, and may even represent a marker of vascular inflammation^8^. As EAT is present in all human anatomy and differentially distributed around the myocardium, it is possible that the activity of EAT may have a greater effect on cardiac and coronary dysfunction. Statins are well described pleiotropic agents that have anti-inflammatory properties^23^. Interestingly, we did not demonstrate any difference in mean EATd at baseline or follow-up by statin stratification with a global reduction in EATd across the entire cohort. This finding may be due to EATd representing a mean attenuation of the totality of EATv and there may be influence on regional EAT differences or on pericoronary adipose tissue attenuation, a novel proposed imaging biomarker of vascular inflammation^24^. A higher adipose tissue attenuation (less negative HU on CT) is suggestive of inflammation due to arrest of lipid maturation^25^. Therefore, another explanation for our findings is the symptom status of patients: baseline CTCA was performed in symptomatic patients whereas the follow-up scan was a research study performed when patients were stable. The lower attenuation values may therefore represent vascular stability and there are several studies that suggest a dynamic response in adipose tissue attenuation^26–28^. One recent sub-study of serial CT evaluation in asymptomatic statin prescribed patients demonstrated an overall lowering of EATd independent of lipid lowering on follow-up, but there was no comparative control group, density was measured in a single region of interest and EATv was not described on follow-up scans^29^. Given the increasing reports of cardiac adipose tissue and its effect on high risk plaque presence, plaque progression and mortality^25^, further investigation of the behaviour and measurement of the activity of these fat depots is needed to translate these results into clinical practice.

While higher volumes of EATv are considered to be pathologic in their associations with cardiac disease, it is not certain whether changes in EATv associate with disease. It is possible that EAT increased as a function of age. EATv has been described to increase in parallel to changes in LV mass on necropsy assessment^30^. LV mass significantly increases with age^31^ and it is possible that changes in LV mass may account for the increase in EATv. Further analysis of this factor as well as association with any progression of coronary disease or cardiac dysfunction are required. In addition, there are no endorsed population thresholds for EATv or EATd that associate with disease, and numerous cut-points relevant to selective cohorts have been described in the literature. As the purpose of this study was purely an observational assessment of the natural history of EAT parameters, it is not clear whether these changes may result in disease or whether change in coronary atheroma was evident. Again, this remains a subject for further assessment.

We acknowledge several limitations in our study. Firstly, this is a small cohort of highly selected patients with only minimal or mild coronary artery disease and therefore our results may only apply to similar patient populations. Secondly, we did not evaluate continuous markers of cardiovascular risk factors such as bloods pressure levels, cholesterol profile indices or HbA1c levels and instead used binary variables of presence or absence of pathology – this was in part related to incomplete information from baseline patient data and may explain the lack of significant correlation found with baseline risk factors which has been previously well described^32^. Thirdly, we only evaluated clinical risk factors that were forced into a multivariable model and cannot account for other potential mediators such as ethnicity, coronary artery disease extent and severity or other medical therapies, however, this is reflective of the current literature in examining relevant associations of EAT. Finally, there is potential for error in using delta EAT values with potential overlap from test-retest variability. Our previous work has demonstrated limits of agreement up to 10 mL higher or lower between observers with a mean bias however of only 1 mL, however our inter-observer correlation was excellent at 0.98 with assessors blinded to scan timing and patient details.

## Conclusion

Epicardial adipose tissue volume and density demonstrate significant longitudinal changes in patients with non-obstructive coronary artery disease with a consistent increase in EAT volume and consistent decrease in EAT density. There are no clinical risk factors that appear to associate with the change in EAT parameters and this effect is also independent of statin therapy. This finding may suggest that EAT is an independent marker, rather than surrogate of cardiovascular risk.

### Data availability

The datasets generated during and/or analysed during the current study are available from the corresponding author on reasonable request.
